# Quality of Machined Surface and Cutting Force When Milling NiTi Alloys

**DOI:** 10.3390/ma17246122

**Published:** 2024-12-14

**Authors:** Małgorzata Kowalczyk, Krzysztof Tomczyk

**Affiliations:** 1 Faculty of Mechanical Engineering, Cracow University of Technology, Jana Pawła II 37 Avenue, 31-864 Krakow, Poland; 2 Faculty of Electrical and Computer Engineering, Cracow University of Technology, Warszawska 24, 31-155 Krakow, Poland; krzysztof.tomczyk@pk.edu.pl

**Keywords:** milling, NiTi alloy, helix angle, cutting force, surface roughness

## Abstract

The machining of shape memory alloys, such as NiTi, presents challenges due to their specific physical, chemical, and mechanical properties. This study investigated the effect of the helix angle of milling tools—both uncoated and coated—on the cutting forces and the surface roughness of the milling process for a NiTi alloy. Experiments were conducted using the tools with and without coatings at various helix angles (20°, 30°, and 40°) and under different machining conditions. Optimization of the process was employed the Taguchi method to identify the best combination of the corresponding parameters. The results of the cutting force and the surface roughness measurements were analyzed and discussed in the context of optimizing the cutting conditions to achieve the desired outcomes. The results show that the lowest surface roughness values (*Sa* = 0.301 μm and *Sz* = 3.41 μm) were achieved with the coated tool at a helix angle of 30°, a feed per tooth of 0.02 mm, and a cutting speed of 45 m/min, while the lowest cutting force (*F* = 143.6 N) was observed with the coated tool at a cutting speed of 55 m/min, helix angle of 40°, and feed per tooth of 0.02 mm. This research provides valuable insights for industrial applications requiring the precise machining of NiTi in terms of the cutting forces and the surface quality. The findings reveal that the presence of the coating, along with an increase in the helix angle, significantly reduces the cutting forces, positively influencing the quality of the machined surface.

## 1. Introduction

NiTi-based shape memory alloys (SMAs) have garnered significant attention in recent years due to their remarkable characteristics, such as the shape memory effect, superelasticity, and impressive corrosion resistance [1,2]. These alloys, capable of returning to their original form after deformation, stand out for their flexibility and durability, making them suitable for numerous high-demand applications. As a result, NiTi alloys have found widespread use in advanced fields, including mechanical engineering, healthcare, and technologies related to the aerospace and automotive sectors [3]. Their high biocompatibility, exceptional strength-to-weight ratio, and outstanding fatigue resistance further boost their appeal for applications like medical implants, orthodontic devices, and smart actuators [4].

Nevertheless, machining of NiTi alloys remains a significant challenge due to their inherent high plasticity, poor thermal conductivity, and the tendency to undergo rapid work hardening during cutting [5,6]. These material properties result in faster tool wear, higher cutting forces, and poorer surface finishes on machined components [7,8]. Low thermal conductivity of NiTi impacts its heat dissipation during the machining process, which can accelerate the degradation of the cutting tool and reduce its efficient life. Additionally, continuous chip formation and burr generation complicate the control over the machining process and lead to suboptimal surface quality. The strength of the material also places high mechanical stress on the tool during cutting, which contributes to increased adhesion of the workpiece material to the tool, leading to faster wear [8,9] and subsequently deterioration of the surface finish. To overcome these challenges, it is crucial to optimize the machining parameters, choose the appropriate tool materials, and employ effective cooling methods to achieve precision and high efficiency while preserving the material’s integrity [10,11].

There are many studies in the literature on the effect of different machining parameters on cutting forces and surface roughness when milling difficult-to-machine NiTi alloy. Altas E. et al. [12,13] analyzed the effects of cutting speed, feed rate, and cryogenic heat treatment on surface roughness and tool wear. Their results indicated that the optimum surface quality with the lowest roughness was achieved at a cutting speed of 50 m/min, a feed rate of 0.03 mm/tooth, and cryogenic cooling, which significantly reduced tool wear.

Kaya E. et al. [14] investigated the influence of the cutting tool material and the cutting speed on the functional integrity of NiTi. Their results showed that cutting speeds above 100 m/min improved the surface quality and reduced the transformation enthalpy and microhardness, resulting in less material damage.

Wang G. et al. [15] focused on the effects of cutting parameters such as cutting speed and the feed rate on surface roughness and work hardening during NiTi milling. Their results showed that increasing the cutting speed reduces the surface roughness, while the combined effect of the speed and feed on hardening depends on the selected parameters.

Gao L. et al. [16] found that increasing the depth of cuts results in greater surface hardness and residual surface topography, which affects the frictional and biocompatible properties of NiTi components.

Zailani Z.A. et al. [17] analyzed the effect of a minimal quantity of lubrication and air-cooling conditions on surface quality and tool wear. The results indicated that cooling reduced tool wear and burr formation, which had a positive effect on the surface quality while allowing modification of NiTi properties through controlled phase transitions.

Wang H. and colleagues [18,19] used the DAM method to create a nanoporous layer on an NiTi surface, resulting in a 14% reduction in cutting forces and 12% reduction in temperature. The nanoporous layer changes in the machining characteristics led to a more brittle nature, reducing tool wear and improving surface quality.

Kaynak et al. [20] developed a simulation model to analyze the phase transformation during machining of NiTi alloy. The study showed that increasing the cutting temperature during machining reduces the martensitic content near the surface, which allows better control of the mechanical properties of the material and reduces the cutting forces.

Shizuka H. et al. [9] investigated the challenges associated with machining NiTi, noting that high cutting forces and temperatures lead to severe tool, wear and the adhesion of superelastic chips to the tool significantly complicates the process.

Kaya E. and Kaya I. [21] conducted a review of research on NiTi machining, highlighting specific challenges such as high cutting forces and tool wear, and emphasizing the importance of machining parameters and conditions for the post-machining functionality of NiTi. These studies clearly show that the selection of appropriate cutting parameters, such as cutting speed, helix angle, feed per tooth (*f_z_*), and effective cooling and lubrication, is critical to optimizing the cutting forces and the surface roughness in NiTi milling while maintaining the desired mechanical properties of the alloy.

A practical solution to improve the machinability of a hard material is to simultaneously optimize the machining parameters and tool geometries in terms of the cutting force and surface roughness. In the context of the milling process of difficult-to-cut machine materials, there are a number of studies that have optimized the cutter helix angle to improve the machinability.

Research by Wang M. et al. [22] involved a theoretical analysis of the effect of the helix angle on peak cutting forces in peripheral milling. They showed that a larger helix angle reduces the peak cutting forces by reducing the load on each cutting edge. As a result, the optimum helix angle that minimizes the cutting forces depends on the factors such as the depth of cut, number of flutes, and cutter diameter.

In research caried out by Sur G. et al. [23], it was experimentally confirmed that the helix angle has a significant effect on the cutting forces and surface roughness, with larger angles improving the surface quality and reducing the cutting forces. Using tools with a high helix angle for Ti6Al4V alloy reduced the average cutting force by 34.71% and reduced the surface roughness by 276.32%.

Research by Tien D.H. et al. [24] showed that the optimum surface roughness for C45 steel was achieved at a helix angle of 45° with appropriate cutting parameters. Increasing the helix angle of the cutter under high-speed cutting conditions had a positive effect on the surface quality.

In a paper by Plodzien M. [25] and others, the effect of the helix angle on the high-speed milling process for the alloy AlZn5.5MgCu was analyzed. It was shown that increasing the angle to 50° reduces the cutting forces and improves chip evacuation, thus increasing cutting efficiency.

Experiments on MgO–WF (magnesium oxide and wood fiber composite) described in a paper by Jiang R et al. [26] showed that a larger helix angle (from 20° to 50°) reduced the cutting forces and improved the surface quality by reducing the roughness and tool wear. In contrast, research by Zagórski I. et al. [27] using AZ91D and AZ31 magnesium alloys showed that tools with the helix angle of 20° gave the best surface quality in finished milling due to increased machining stability.

In addition, Joshi SN et al. [28] investigated the effects of 35° and 55° helix angles on the milling of 2024-T351 aluminum alloy. The results indicated that a larger helix angle (55°) reduced the cutting forces and improved the surface integrity, which is particularly important when the machining thin-walled, low-stiffness parts.

In research by Tomáš Knápek et al. [29] on the effect of the clearance angle on the cutting forces and surface roughness when milling carbon fiber-reinforced composites (CFRP), it was found that a smaller clearance angle leads to faster tool wear, higher cutting forces, and lower surface quality. The experiment compared three different clearance angles (8.4°, 12.4°, and 16.4°), and the results suggest that the smaller the clearance angle, the greater the cutting forces and surface roughness. The tool with an 8.4° clearance angle generated higher cutting forces and wore out faster than the tool with a 16.4° clearance angle, resulting in greater surface roughness and more edge damage to the material.

Despite comprehensive research on the role of the milling cutter helix angle on the machinability of hard material, the impact of this parameter and its interaction with other machining factors in milling of NiTi have hardly been investigated in the literature. In particular, there is a lack of studies investigating how variations in helix angle affect the cutting forces and surface roughness specifically for NiTi, and how these effects differ between coated and uncoated tools. Such comparisons are essential to better understand the unique challenges of machining NiTi and to optimize the process for both performance and efficiency. Therefore, the machinability of this material in the milling process merits further investigation. To address the challenge regarding machining of NiTi in milling kinematics, this study was designed to optimize the machining parameters using the Taguchi approach (Table 1). Nine experiments (Table 2) based on the L_9_ orthogonal array were designed to identify the effects of the helix angle, cutting speed, and feed rate on the surface roughness and cutting forces. The objective of the study was to assess the contribution of each factor to the cutting force *F* and surface roughness (*Sa*, *Sz*). Subsequently, the influence of the processing factors on these responses was analyzed, with consideration given to the physical mechanisms underlying the cutting process and the milling kinematics. Finally, an optimal solution was identified using the Taguchi method, with the aim of simultaneously minimizing both the surface roughness and cutting force. In this study, the cutting force and surface roughness were selected as evaluation indicators due to their critical role in assessing both machinability and the quality of the machined surface. Previous research showed that the cutting forces provide an essential insight into the energy and effort required during machining. This is evidenced by studies such as that of Yin et al. [30], which highlights the importance of the cutting forces in assessing the machinability of difficult materials such as Inconel 718. In addition, surface roughness is a key parameter in surface integrity and has a direct influence on the functionality and aesthetics of the final product, as demonstrated by research investigating the relationship between surface roughness and various machining conditions [31,32].

## 2. Materials and Methods

**Figure 1:**
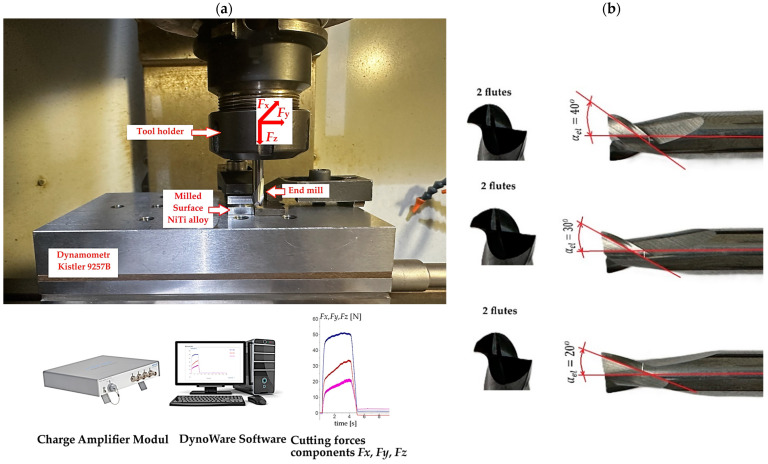
(**a**) Experimental setup for milling NiTi, $F_{x}\;$—blue color, $F_{y}$—red color, $F_{z}\;$—pink color; (**b**) the three tools with different cutter helix angles.

A three-axis CNC vertical center equipped with a Haas control system (Haas VF1) was used to conduct the experiments on the NiTi alloy workpiece using the experimental setup shown in Figure 1a. The material used in this work was a NiTi shape memory alloy, which was slightly off-stoichiometry with 58.01 Ni (wt.%), 41.99 Ti (wt.%), obtained from Baoji Hanz Metal Material Co., Ltd. (Baoji, China). The austenite finish temperature was $A_{f}=60\;{}^{\circ}C$. Three AlTiN-coated and uncoated carbide end mills were applied: two flat-end milling cutters with diameters of *D* = 6 mm, a tool rake, and clearance angles of γ=12° and α=6°. However, the helix angles of the tools varied: 20°, 30°, and 40°. Figure 1b shows the geometric characteristics of the flat solid carbide end mills used for the study. The process was carried out using down milling kinematics with varied cutting velocity and feed per tooth in the range of ${\;v}_{c}=35-55\;m/min$ and feed per tooth $f_{z}=0.02-0.06\;mm/tooth$. The depth and width of cuts were kept constant at $a_{p}=4\;mm$ and $a_{e}=0.4\;mm$, respectively. It needs to be added that all the experiments were peformed under dry machining conditions. The helix angles of 20°, 30°, and 40°, as well as the cutting speed and feed rate, were selected based on a review of the relevant literature, the authors’ experience in machining NiTi alloys, and preliminary studies. These parameters reflect common practice and are tailored to the capture the specific behavior of this material during machining. The experiments were designed based on the L9 Taguchi orthogonal array incorporating the three factors at three levels, as shown in Table 1. Table 2 shows the design matrix of the nine sets of experiments. Widely utilized in engineering analysis, the Taguchi method emphasizes controlled experimental design to obtain valuable insights into the behavior of a given process [12,13,33]. The primary advantage of this method lies in its efficiency, significantly reducing the number of experiments required, saving time, cutting costs, and quickly identifying critical factors. The Taguchi optimization steps applied in this study were as follows: select the noise and control factors, determine the suitable working levels of the design factors, select the Taguchi orthogonal array, run experiments, roughness parameters and cutting force measurement, analyze results (signal-to-noise ratio), and predict optimum performance.

Three components of the milling cutting force ($F_{x},\;F_{y},\;F_{z}$) were measured using a Kistler 9257B (Kistler Group, Winterthur, Switzerland) piezoelectric dynamometer (Figure 1). Visualization, processing, and saving of the signal were carried out using DynoWare version 3.1.2.0 software. The sampling frequency of the signal was set to 7 kHz and each measurement was repeated three times to ensure reliability and reproducibility of the results.

**Figure 2:**
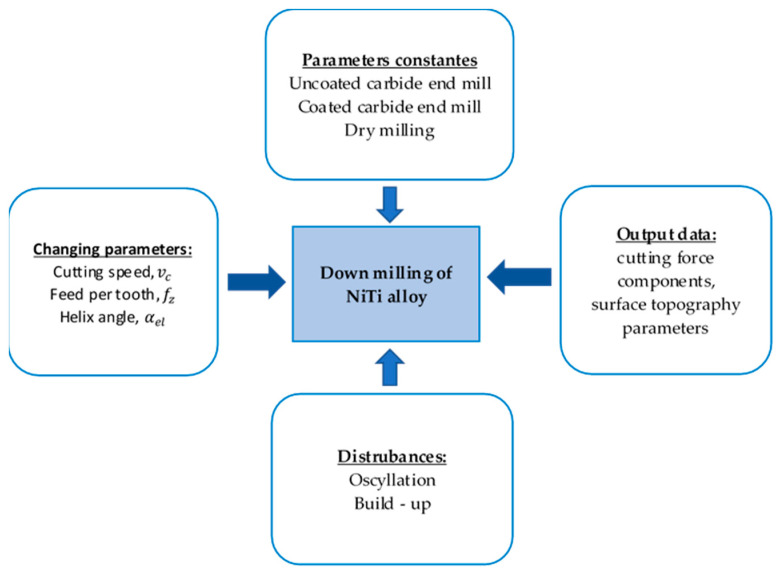
Schematic diagram of the test setup.

The machined surface roughness was measured using a contact surface profilometer (Taylor Hobson, Leicester, UK). The TalyMap program was used to visualize the surface test measurements. Measurements were carried out at various locations on the machined surface, with each location measured three times and the average value of these repeated measurements used for further analysis. Post-processing of the raw data included noise removal, shape profile filtering, topography imaging with 3D maps, and determination of the selected surface topography parameters. The surface topography parameters were determined according to ISO25178. The choice of the tools was determined by examining the influence of the tool helix angle (rake angle) on the surface roughness and cutting force component. A schematic diagram of the test setup is shown in Figure 2.

## 3. Analysis of the Results

**Figure 3:**
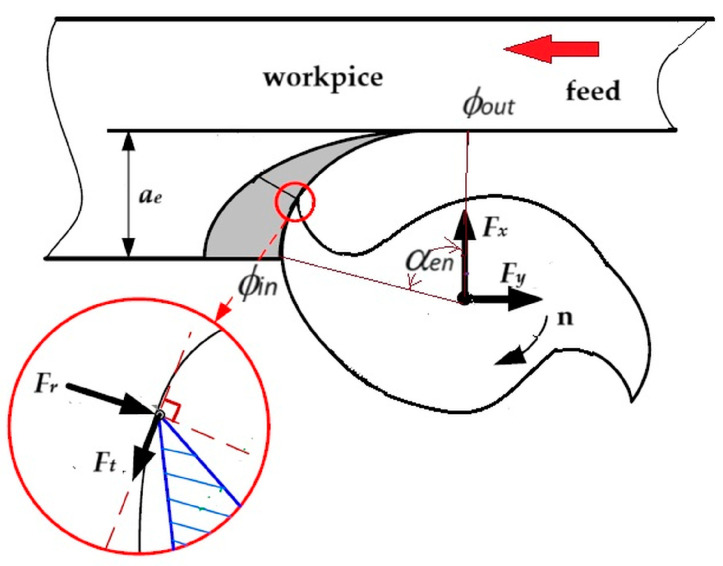
Schematic representation of the cutting force component in the tool system, where $F_{t}\;$(N)—tangential cutting force, $F_{f\;}$ (N)—feed force $F_{r}\;$(N)—radial cutting force, $a_{e}$ (mm)—the radial depth of cut; $\emptyset_{in}$ (rad)—entry angle, $\emptyset_{out}$ (rad)—exit angle, $\alpha_{en}$ (rad)—radial engagement angle, n (rev/min)—spindle speed.

Monitoring and controlling the cutting forces when milling NiTi shape memory alloys is critical to optimizing the quality of the machined surface and improving cutting tool performance [11]. When machining difficult-to-machine alloys, such as NiTi alloys, a significant resultant cutting force acts on the cutter edge. This force *F* is the result of a combination of several components, which are generated as a result of the contact between the tool and the difficult-to-machine material. The resulting cutting force *F* leads to intensive wear of the cutting edge, which in turn can reduce the machining accuracy and tool life. A schematic representation of the cutting force component in the tool system is shown in Figure 3.

Following the completion of the machinability tests and cutting force and surface roughness measurements, the initial stage of the process involved the single-objective optimization and evaluation of the control factors/process parameters that were identified as providing the minimum cutting force and surface roughness values. This was conducted using the Taguchi method. The research results were visualized using Minitab 22.

In order to the model the cutting forces for end milling, a mechanistic force model was applied, which has proven to be effective in previous studies [11,34,35]. This model relates the cutting force components to the undeformed chip thickness. At a given time point *t*, tangential ($F_{t}$) and radial ($F_{r}$) forces are applied to the k-th flute of a rigid end mill. These forces can then be resolved into the feed ($F_{y}$) and transverse ($F_{x}$) force components as follows:

(1) $$F_{t}=F_{y}cos\theta +F_{x}sin\theta$$

(2) $${\;F}_{r}=F_{x}cos\theta {-F}_{y}sin\theta$$

In down milling, the shape of the cutting force for a single-flute end mill is dependent upon both the cutter engagement angle $\alpha_{en}$ and the cutter swept angle $\alpha_{sw}$. These angles are in turn related to the cutting parameters [11].

The engagement angle $\alpha_{en}\;$ is defined in terms of the radial depth of cut *a_e_* according to the following equation:

(3) $$\alpha_{en}=\emptyset_{out}-\emptyset_{in}=arccos(1-\frac{a_{e}}{D})$$

The axial engagement angle $\alpha_{sw}$ is related to the axial depth of cut *a_p_* with the following equation:

(4) $$\alpha_{sw}=\frac{2\tan(\alpha_{el})}{D}a_{p}$$

where *D* is the tool’s diameter, *a_p_* is the axial depth of cut, *a_e_* is the radial depth of cut and $\alpha_{el}=20^{{}^{\circ}},\;30^{{}^{\circ}},\;40^{{}^{\circ}}$ are the cutter’s helix angle parameters.

It can be demonstrated that $the\;angle\;\theta =\alpha_{en}+\alpha_{sw}$ represents the angular positions where the cutting edge is fully engaged in the cutting process and thus identifies the maximum cutting force [11].

Table 3 presents the results of measurements of the maximum cutting force components during milling of the NiTi alloy within the adopted range of values tested.

**Figure 4:**
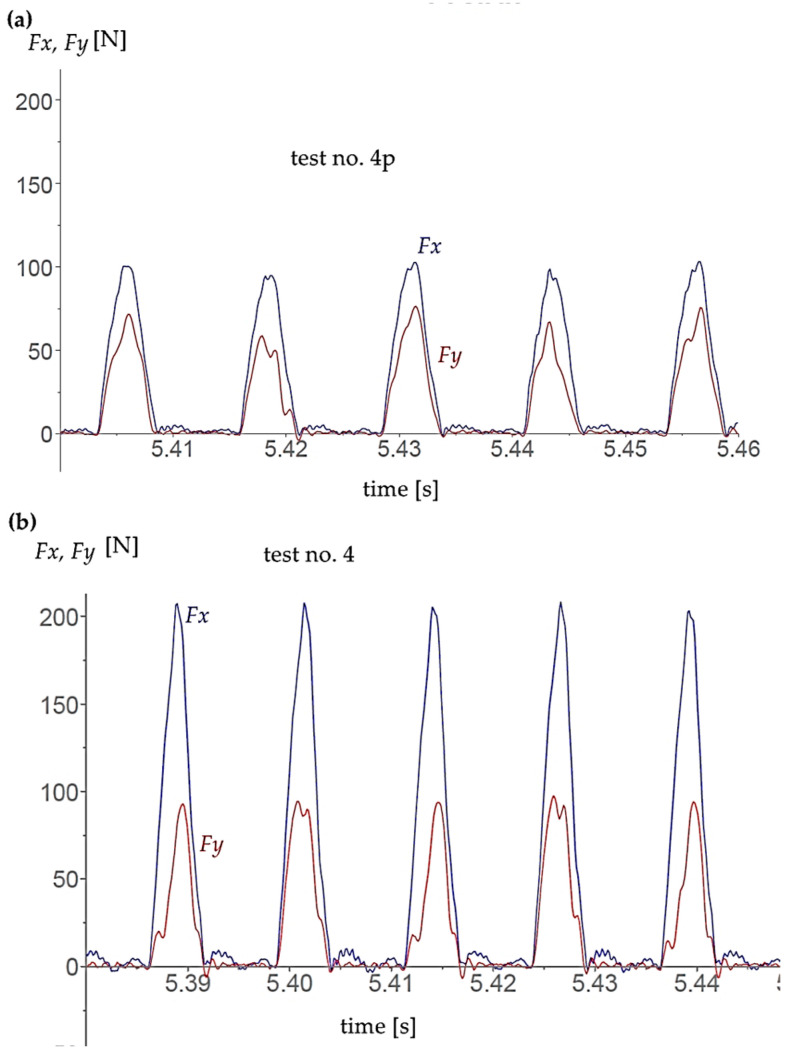
Details of the cutting force record for two flutes for (**a**) coated tool and (**b**) uncoated tool for cutting parameters $v_{c}=45\frac{m}{\min},f_{z}=0.02\frac{\mathrm{mm}}{\mathrm{tooth}},\;\alpha_{el}=20^{{}^{\circ}}$.

Figure 4 illustrates the presentation of results in Kistler’s DynoWare version 3.1.2.0 software. The blue indicates the loading on the X-axis, while the red represents the loading on the Y-axis. The graph displays the work of individual flutes. The measured forces that can be observed between engagements are caused by vibrations.

In the Taguchi method, the term “signal” denotes the optimal value (mean) for the output characteristics, whereas the term “noise” signifies the suboptimal value for the aforementioned characteristics [36]. The Taguchi method employs the S/N ratio as a means of assessing the quality characteristic that deviates from the desired value. There are several S/N ratios that may be employed, contingent on the type of characteristics under consideration. These include the ratios of lower is better (LB), nominal is the best (NB), and higher is better (HB) [13]. In this study, the smaller-is-better S/N ratio was employed, as the lower parameters of the surface roughness (*Sa* and *Sz*) and cutting force *F* were deemed favorable [5]. The quality characteristics of “smaller is better” are calculated according to the following equation [12]:

(5) $$S/N=-10\times \log_{10}\times [(1/n)\times \sum \left(y_{i}^{2}\right)]$$

where *n* is the number of measurements in a trial/row and *y_i_* is the measured value in a run/row.

Table 3 presents the S/N ratio values for the total cutting force acting on the uncoated and coated milling cutter flute obtained for the different parameter levels and in accordance with the “smaller is better” criterion.

**Figure 5:**
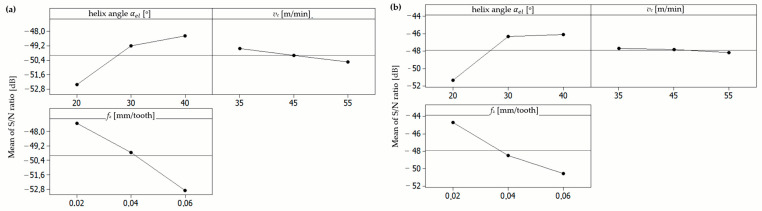
S/N ratio values for parameter *F:* (**a**) uncoated mill; (**b**) coated mill.

In the case of the uncoated milling cutter (Figure 5a, Table 3), the highest S/N values, which correspond to the lowest cutting force, were observed at the following parameter combinations: test 7 (cutting speed of 55 m/min, feed per tooth of 0.02 mm/tooth, and helix angle of $40^{{}^{\circ}}$) with an S/N value of −46.01 for the uncoated mill. Similarly, for the coated mill (Figure 5b, Table 3), the highest S/N ratios were identified for test 7 (cutting speed of 55 m/min, feed per tooth of 0.02 mm/tooth, and helix angle of $40^{{}^{\circ}}$) with an S/N of −46.21. After the application of a single-criterion optimization methodology based on the Taguchi method, with the objective of minimizing the cutting force, it can be concluded that the optimal set of parameters is as follows: cutting speed of 55 m/min, feed per tooth of 0.02 mm/tooth, and helix angle of $40^{{}^{\circ}}.$

**Figure 6:**
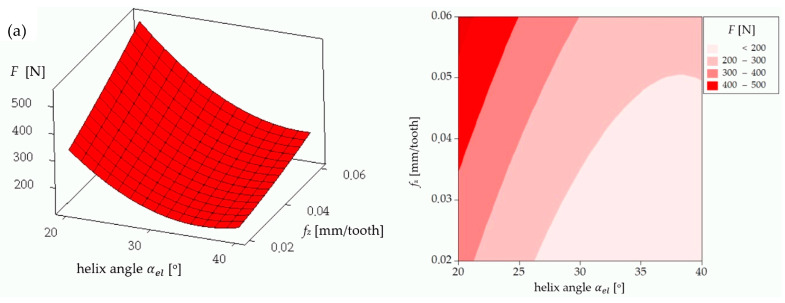

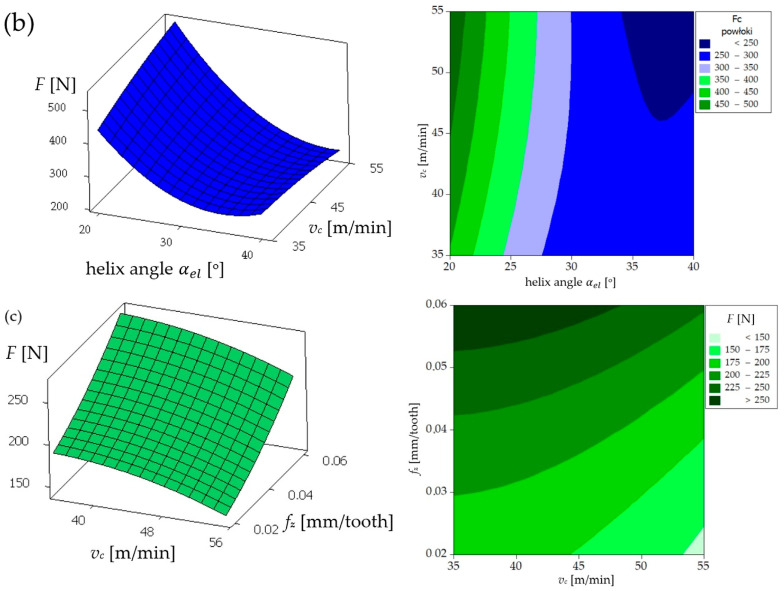
Cutting force *F* for a single flute for the coated mill: (**a**) influence of helix angle and feed per tooth on cutting force (*F*) for cutting speed of 55 m/min; (**b**) influence of helix angle and cutting speed on cutting force (*F*) for feed of 0.06 mm/tooth; (**c**) influence of feed per tooth and cutting speed on cutting force (*F*) for helix angle of $40^{{}^{\circ}}$.

The analysis of the influence of the helix angle ($\alpha_{el}$), feed per tooth (*f_z_*), and cutting speed (*v_c_*) on the main cutting force (*F*) acting on a coated milling tool provides a valuable insight into the optimization of the machining process. As illustrated in Figure 6a, there is a notable increase in the cutting force (*F*) with an increase in the feed per tooth. This phenomenon can be attributed to the increased volume of material removed per tooth engagement, which exerts a greater load on the cutting edge and consequently increases the resistance force acting on the tool. Furthermore, Figure 6a illustrates that the helix angle $(\alpha_{el})\;$ exerts an inverse influence on the cutting force. As the helix angle increases, the cutting force *F* declines. This can be attributed to the fact that a larger helix angle allows for a more gradual engagement of the cutting edge with the material, which distributes the cutting load more evenly along the cutting edge, thereby reducing the resistance and resulting in a lower cutting force. Figure 6b provides further illustration of the impact of the helix angle and the cutting speed on the cutting force. As the cutting speed (*v_c_*) increases, a decrease in the cutting force (*F*) is observed. This phenomenon can be attributed to the thermal softening of the workpiece material at higher cutting speeds. Higher speeds generate increased heat, which reduces the material’s yield strength, thereby facilitating cutting and consequently reducing the cutting force. Furthermore, Figure 6b corroborates that a larger helix angle results in a reduction in the cutting force. Figure 6c provides further elucidation of the relationship between feed per tooth and cutting speed on cutting force. As previously indicated in Figure 6a, the cutting force is observed to increase with higher feed per tooth. Moreover, an increase in the cutting speed was observed to result in a reduction in the cutting force, which is consistent with the findings presented in Figure 6b.

In conclusion, the findings demonstrate that the feed per tooth (*f_z_*) has the most significant influence on enhancing the cutting force *F*, with elevated feed per tooth (*f_z_*) values leading to augmented tool loading. Conversely, an increase in the helix angle ($\alpha_{el}$) and cutting speed (*v_c_*) has the effect of reducing the cutting force. A larger helix angle allows for a more efficient distribution of the cutting load along the cutting edge. Conversely, higher cutting speeds reduce the material strength due to the thermal effects, thus lowering the cutting resistance. The aforementioned analysis, conducted for a coated milling tool, demonstrates that selecting a larger helix angle and higher cutting speed, while carefully managing the feed per tooth, can enhance the efficiency of the cutting process by reducing the cutting force acting on the tool. Furthermore, the coating on the tool contributes to this efficiency by reducing the friction and wear, allowing the tool to withstand higher cutting parameters while maintaining lower cutting force.

**Figure 7:**
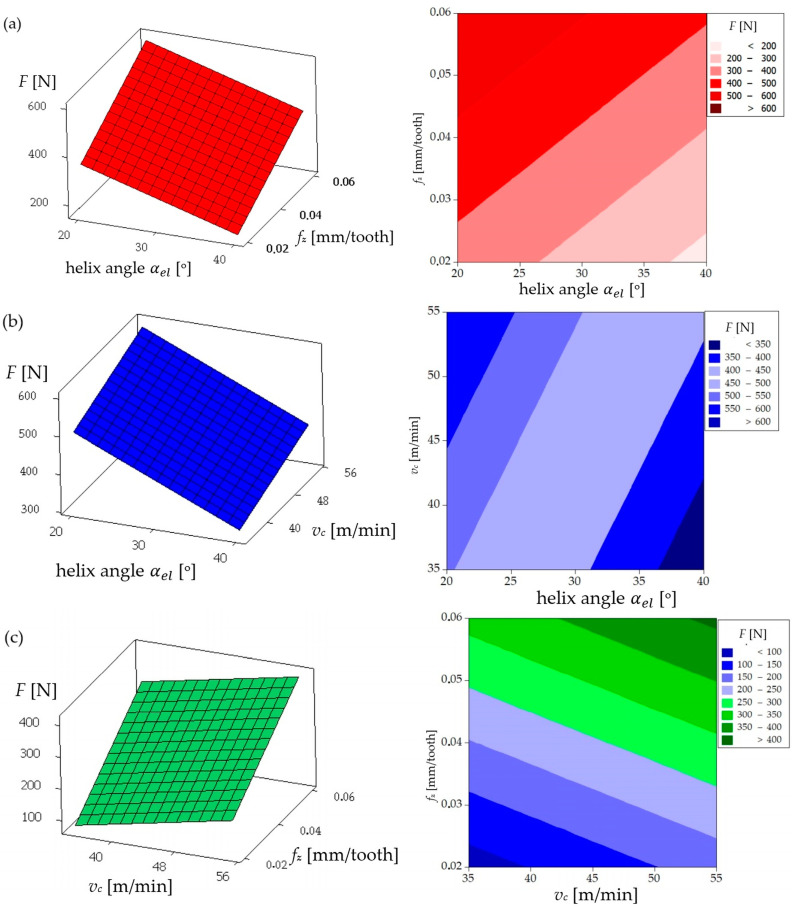
Cutting force *F* for a single flute for the uncoated mill: (**a**) influence of helix angle and feed per tooth on cutting force (*F*) for cutting speed of 55 m/min; (**b**) influence of helix angle and cutting speed on cutting force (*F*) for feed per tooth of 0.06 mm/tooth; (**c**) influence of feed per tooth and cutting speed on cutting force (*F*) for helix angle of $40^{{}^{\circ}}$.

The analysis of the influence of the helix angle ($\alpha_{el}$), the feed per tooth (*f_z_*), and the cutting speed (*v_c_*) on the main cutting force (*F*) acting on an uncoated milling tool provides valuable insights into how a tool without coating affects the milling process of the NiTi alloy. A comparison of the aforementioned results with those obtained for the coated tool reveals significant differences in the behavior and the magnitude of the cutting force, which serves to underscore the impact of tool coating on the machining process. Figure 7a illustrates that for the uncoated tool, the cutting force *F* increases with an elevated feed per tooth (*f_z_*), in a manner analogous to the observations made for the coated tool. This phenomenon can be explained by the fact that a larger feed per tooth results in a greater quantity of material being removed with each pass of the tool, which consequently increases the load on the cutting edge and subsequently the resistance force acting on the tool. The helix angle ($\alpha_{el}$) exerts an inverse influence on the cutting force *F* to feed per tooth, with an increase in the helix angle leading to a reduction in the cutting force *F*. Figure 7b illustrates the impact of the helix angle and the cutting speed on the cutting force for the uncoated tool. As illustrated in Figure 7b, an increase in the cutting speed results in an increase in the cutting force when using the uncoated tool. The increase in the cutting force at higher cutting speeds when machining NiTi alloy with an uncoated cutter can be attributed to the fact that the cutter blade comes into contact with the workpiece with higher kinetic energy. The higher cutting speed increases the intensity of the thermal and mechanical load on the cutting edge, which results in the workpiece material exhibiting increased hardness and resistance to plastic deformation.

Following an investigation into the impact of the cutting parameters and the helix angle on the cutting force *F* for the coated tool, an ANOVA was conducted. The results demonstrated that the helix angle (*F* = 11.76, *P* = 0.019) and the feed per tooth (*F* = 11.59, *P* = 0.019) exerted a statistically significant influence on the cutting forces. Nevertheless, the cutting speed (*F* = 1.15, *P* = 0.332) did not demonstrate a statistically significant effect. Conversely, the findings for the uncoated tool indicate that the helix angle (*F* = 6.81, *P* = 0.048) and the feed rate (*F* = 10.77, *P* = 0.022) also had a statistically significant impact on the cutting forces. Nevertheless, in a manner analogous to the coated tool, the cutting speed (*F* = 1.69, *P* = 0.25) did not exhibit a notable impact.

A comparison of the cutting force values for the uncoated and the coated tools revealed a notable distinction: the uncoated tool exhibited considerably higher cutting forces across all parameter ranges. This is particularly evident in the color gradients on each contour plot, which demonstrate that the force range for the uncoated tool is generally higher. The coating on the tool serves to reduce the friction between the tool and the workpiece, which in turn results in a reduction in the cutting force required to perform the cutting operation. The uncoated tool, lacking the protective layer, experiences higher friction and consequently higher cutting forces.

In conclusion, while the trends in the effect of the helix angle and the feed per tooth on the cutting force remain consistent between the coated and uncoated tools, the absolute values of the cutting force are substantially higher for the uncoated tool. This difference demonstrates the advantages of utilizing a coated tool in the machining operations. The coating reduces the cutting forces, allowing for potentially higher cutting parameters, while maintaining the stable processing conditions.

The results of our study indicate that an increase in the feed rate and the cutting speed is associated with an elevation in the cutting forces, a finding that aligns with those of previous research. A review of the literature on the machinability of hard materials, including Ti-6Al-4V, Inconel 718, and NiTi alloys [2,10,24,35] reveals a consistent finding: higher feed rates result in increased cutting forces. This phenomenon can be attributed to the generation of thicker chips at higher feed rates, which exert greater mechanical loads on the tool, thereby increasing the friction and cutting forces. This correlation between the feed rate and the cutting force was extensively documented across a range of materials.

Similarly, it was observed that elevated cutting forces were also attributable to higher cutting speeds. This trend is consistent with the existing literature, particularly for materials such as Ti-6Al-4V and Inconel 718 [24,35], where increased cutting speeds led to higher thermal loads. The generation of heat at elevated speeds intensifies the wear and friction of the tool, thereby increasing the cutting forces. Concurrently, the elevated temperatures associated with these speeds may diminish friction at the tool–chip interface, potentially enhancing the surface finish. This dual effect highlights the intricate nature of the relationship between the cutting speed and the machining performance.

Conversely, the results demonstrate that an increase in the cutting edge inclination angle resulted in a reduction in the cutting forces. This observation is in accordance with previous findings demonstrating that larger cutting edge angles facilitate improved chip evacuation and reduce the friction between the tool and the workpiece. This in turn reduces the cutting forces, a phenomenon that is well documented in the machining of hard-to-machine materials such as Inconel 718 [35].

### Surface Roughness

The quality of a surface, typically measured by surface roughness, is largely determined by machining process parameters, including cutting tool geometry (e.g., corner radius and rake angle), cutting parameters (e.g., cutting speed, feed, and depth of cut), tool wear, cooling methods, and the material properties of both the tool and the workpiece [7,11]. Surface roughness is a pivotal quality indicator that exerts a considerable influence on the production costs, as it defines the surface geometry, and in conjunction with the surface texture, affects tool performance by influencing the friction and wear. Attaining a target roughness value frequently necessitates an iterative process, necessitating empirical adjustments to reach acceptable levels. The intricate, process-dependent nature of surface roughness formation renders analytical prediction a challenging undertaking, underscoring the necessity for meticulous evaluation during the machining of NiTi alloys [37,38].

Once the machinability tests and the surface roughness measurements were complete, the next stage of the process involved optimizing and evaluating the control factors parameters that were identified as providing the minimum surface roughness values *Sa* and *Sz*. This was conducted using the Taguchi method. Table 4 shows the S/N ratio values for the surface roughness parameters (*Sa*, *Sz*) acting on the uncoated and the coated cutter flute obtained for the different parameter levels and according to the “smaller is better” criterion. The parameters *Sa* and *Sz* were selected for surface roughness evaluation as they provide complementary insights into the quality of the machined surface. While *Sa* represents the average roughness, offering a general measure of surface texture, *Sz* highlights the peak-to-valley differences, which are critical for assessing extreme variations that may affect functional performance. The research results were visualized using Minitab 22 software.

**Figure 8:**
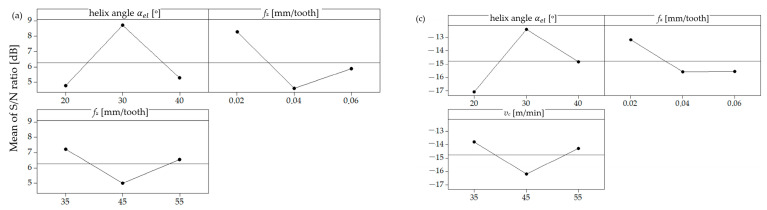

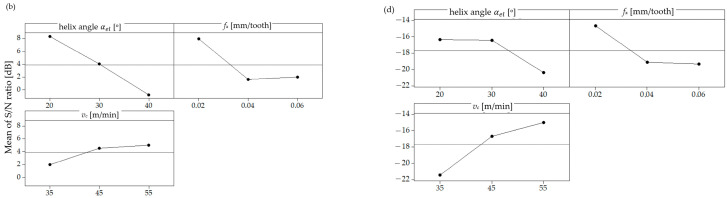
S/N ratio values for parameters *Sa* and *Sz*: (**a**) *Sa* for coated mill; (**b**) *Sa* for uncoated mill; (**c**) *Sz* for coated mill; (**d**) *Sz* for uncoated mill.

According to the Taguchi method’s “smaller is better” criterion, for the surface roughness parameters *Sa* and *Sz*, the highest S/N ratio values should correspond to the lowest roughness values, as a higher S/N ratio indicates more desirable results for minimizing roughness. For the *Sa* parameter of the coated milling tool (Figure 8a), the highest S/N ratio was observed at the helix angle of 30°, feed per tooth of 0.02 mm, and cutting speed of 45 m/min. These settings therefore represent the optimum conditions for minimizing *Sa* roughness with the coated tool. For the uncoated tool (Figure 8b), the maximum S/N ratio for the *Sa* parameter was found at the helix angle of 20°, feed per tooth of 0.02 mm, and cutting speed of 35 m/min, suggesting that these are the ideal settings for achieving the lowest *Sa* values without coating. For the *Sz* parameter in the case of the coated tool (Figure 8c), the optimum configuration—with the highest signal-to-noise ratio—is also achieved at the helix angle of 30°, feed per tooth of 0.02 mm, and cutting speed of 45 m/min. For the uncoated tool (Figure 8d), the maximum S/N ratio for the *Sz* parameter occurred at the helix angle of 40°, feed per tooth of 0.02 mm, and cutting speed of 55 m/min.

In summary, the results indicate that for coated and uncoated tools, a helix angle of 30°–40°, low feed per tooth (0.02 mm), and moderate cutting speed (35–55 m/min) provide the optimum conditions for achieving the lowest surface roughness values for both the *Sa* and *Sz* parameters. This analysis suggests that the highest helix angle and low feed per tooth (*f_z_*) are essential for minimizing the roughness, while the optimum cutting speed may vary slightly depending on the presence of a coating.

The discrepancy in the parameter settings required to achieve minimal surface roughness between the coated and uncoated tools can be attributed to the differing tribological and thermal properties of the tools themselves. Tools coated with AlTiN, for example, demonstrate enhanced wear resistance and reduced friction coefficients, which augment their capacity to sustain a stable cutting process, particularly at elevated cutting speeds. This stability results in a reduction in irregularities in the interaction between the tool and the workpiece, which in turn leads to a minimization of surface defects. Consequently, the optimal surface roughness values are achieved by coated tools at slightly higher cutting speeds (45 m/min for *Sa* and *Sz*).

Conversely, the uncoated tools are devoid of the protective barrier provided by coatings, rendering them more susceptible to wear, adhesion, and heat accumulation at higher speeds. This necessitates the use of lower cutting speeds (35 m/min for *Sa*) in order to achieve a stable cutting process with minimal thermal effects. Furthermore, the optimal helix angle for uncoated tools may vary due to the necessity of efficient chip evacuation and reduced cutting forces in order to compensate for their lower thermal and mechanical performance.

These differences serve to highlight the importance of selecting tool-specific cutting parameters in order to account for the unique material and performance characteristics of coated versus uncoated tools.

The final milling tests of the NiTi alloy, conducted with the use of the coated and uncoated carbide end mills, revealed the influence of the cutting parameters (cutting speed and feed per tooth) and the helix angle ($\alpha_{el}$) on the selected parameters of the surface topography. The results of these tests are presented in Table 4, which also shows the surface topography parameters measured after the milling process.

**Figure 9:**
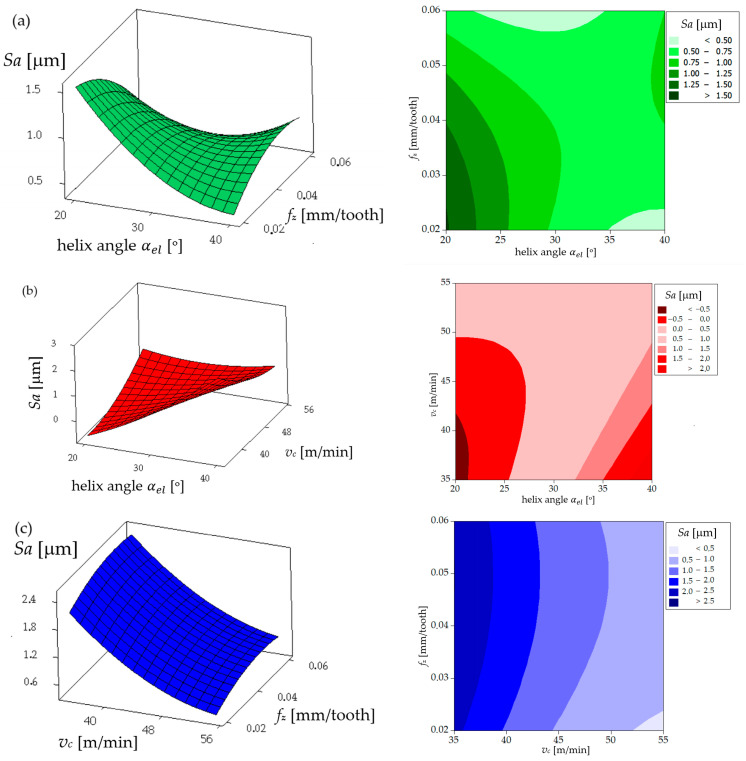
Amplitude *Sa* parameter for the uncoated mill: (**a**) influence of helix angle and feed per tooth on amplitude *Sa* parameter for cutting speed of 55 m/min; (**b**) influence of helix angle and cutting speed on amplitude *Sa* parameter for feed per tooth of 0.06 mm; (**c**) influence of feed per tooth and cutting speed on amplitude *Sa* parameter for helix angle of $40^{{}^{\circ}}$.

Analysis of the influence of the cutting process parameters, including feed per tooth (*f_z_*), helix angle ($\alpha_{el}$), and cutting speed (*v_c_*), on the surface roughness parameter *Sa* for the uncoated tool allows for the assessment of their impact on the quality of the surface finish. As can be observed from the results presented in Figure 9, an increase in feed per tooth (*f_z_*) is associated with an increase in the *Sa* parameter, a relationship that is particularly evident in graphs (a) and (c). The effect of the helix angle ($\alpha_{el}$) on the roughness parameter *Sa* is contingent upon the feed per tooth. As illustrated in Figure 9a, an elevation in the helix angle ($\alpha_{el}$) is correlated with a reduction in the *Sa* parameter. This correlation can be attributed to a more uniform load on the cutting edge of the tool at the larger helix angle ($\alpha_{el}$), which consequently diminishes the roughness amplitude on the machined surface. Conversely, Figure 9b demonstrates that the roughness *Sa* parameter exhibits a slight increase as the cutting speed rises. The impact of the cutting speed (*v_c_*) on the *Sa* parameter is, however, intricate and contingent upon the interplay of other process parameters. At higher cutting speeds, an increase in the *Sa* roughness can be observed, particularly for low feed-per-tooth values and smaller helix angles ($\alpha_{el}$), as evidenced in Figure 9b,c. These dependences findings indicate the necessity for optimization of the feed per tooth and the helix angle ($\alpha_{el}$) of the cutter in order to attain the desired surface roughness, particularly for uncoated tools, where control of *Sa* roughness is vital for the attainment of high surface quality.

**Figure 10:**
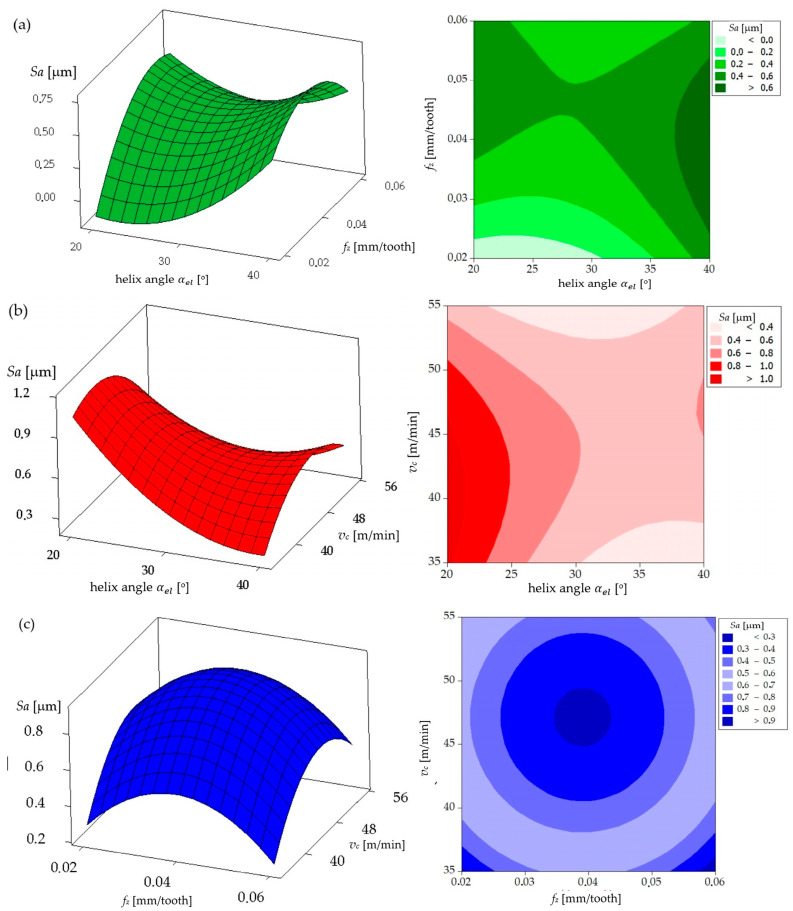
Amplitude *Sa* parameter for the coated mill: (**a**) influence of helix angle and feed per tooth on amplitude *Sa* parameter for cutting speed of 55 m/min; (**b**) influence of helix angle and cutting speed on amplitude *Sa* parameter for feed per tooth of 0.06 mm; (**c**) influence of feed per tooth and cutting speed on amplitude *Sa* parameter for helix angle of $40^{{}^{\circ}}$.

Investigation of the influence of machining parameters, including feed per tooth (*f_z_*), helix angle ($\alpha_{el}$), and cutting speed (*v_c_*), on the surface roughness *Sa* parameter for the coated tools reveals a range of dependences, as illustrated in the graphs and contour maps in Figure 10. An increase in the feed per tooth is observed to result in an increase in the *Sa* values, which is consistent with observations made for uncoated tools. However, in the case of the coated tools, this effect is somewhat less pronounced, especially at lower feed rates, which suggests that the coating may provide protective properties that reduce the friction and tool wear. An increase in helix angle correlates with a reduction in *Sa* values for cutting speed (*v_c_*), while it increases with feed per tooth (*f_z_*). This indicates that the coating facilitates superior roughness control at elevated helix angles, potentially due to the augmented resistance to the adhesion of the machined material. As illustrated in the contour maps, the cutting speed (*v_c_*) exerts a more consistent influence on the *Sa* parameter for the coated tools. This phenomenon may be attributed to the thermal properties of the coating, which mitigate the effects of the friction and the temperature on the surface quality. In the contour map, it can be observed that coated tools permit lower *Sa* values across a more extensive range of parameters in comparison to uncoated tools. This can be attributed to the coating properties, such as a reduced friction coefficient and increased durability, which permit superior maintenance of tool geometry throughout the machining process.

**Figure 11:**
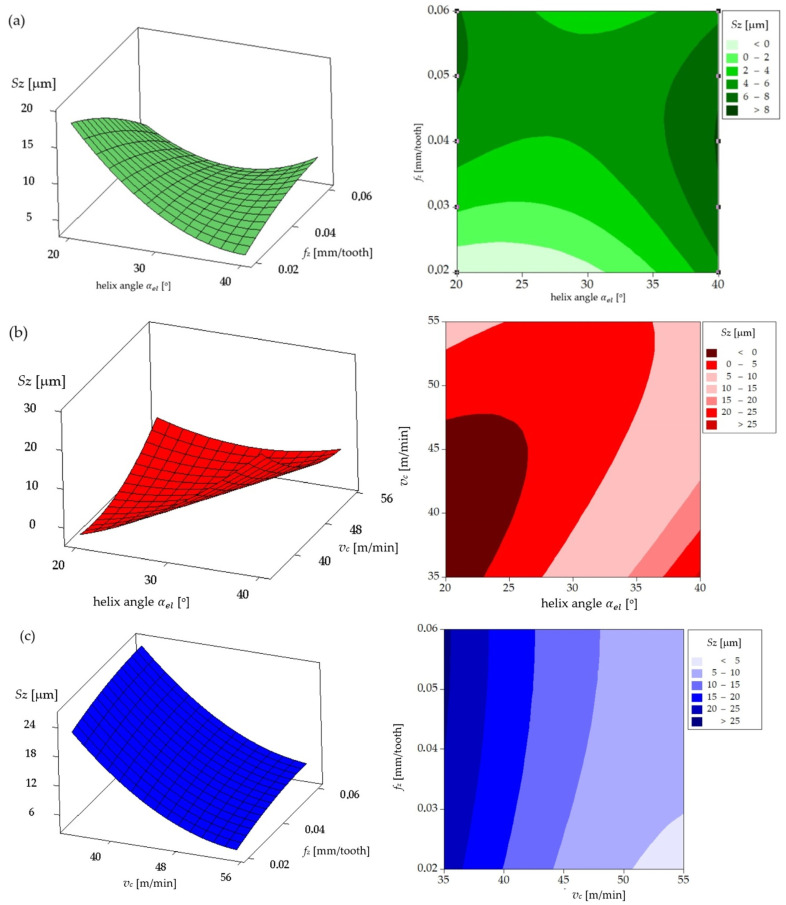
Amplitude *Sz* parameter for the uncoated mill: (**a**) influence of helix angle and feed per tooth on amplitude *Sz* parameter for cutting speed of 55 m/min; (**b**) influence of helix angle and cutting speed on amplitude *Sz* parameter for feed per tooth of 0.06 mm; (**c**) influence of feed per tooth and cutting speed on amplitude *Sz* parameter for helix angle of $40^{{}^{\circ}}$.

In Figure 11a, the surface plot and contour map for the uncoated tool show the influence of helix angle and feed per tooth (*f_z_*) on the *Sz* parameter. Both the surface plot and contour map indicate that lower helix angles and lower feed per tooth (*f_z_*) result in smaller *Sz* values. In contrast, Figure 11b illustrates how cutting speed and helix angle affect the *Sz*: higher cutting speeds lead to higher *Sz* values, especially at lower helix angles, which is visible in the contour map as darker areas. Figure 11c illustrates the impact of cutting speed and feed per tooth (*f_z_*), revealing that increased feed rates and cutting speeds result in higher surface roughness (*Sz*). The contour map in this case also shows a significant increase in *Sz* in the range of higher feed per tooth (*f_z_*) and speeds.

**Figure 12:**
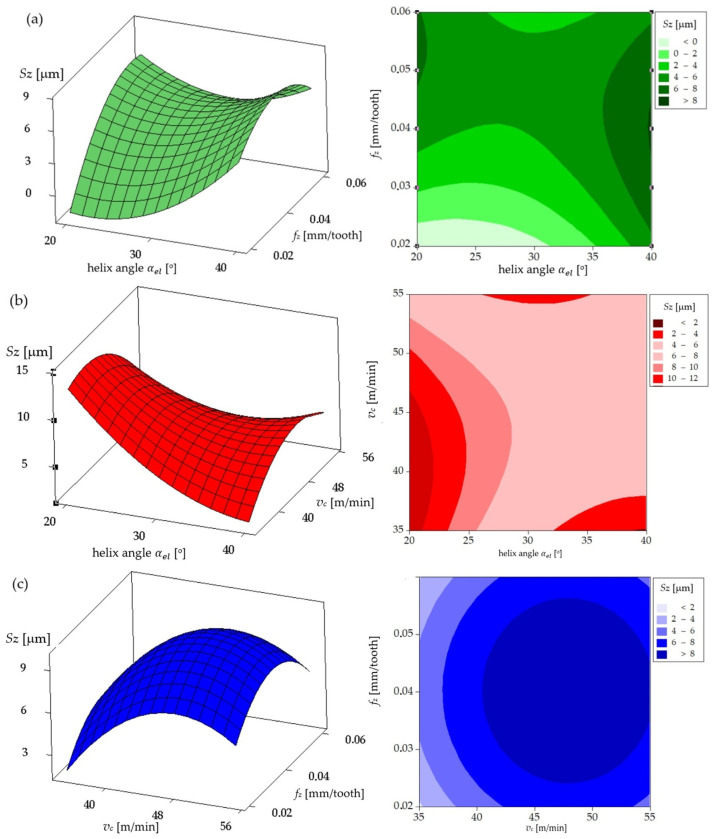
Amplitude *Sz* parameter for the coated mill: (**a**) influence of helix angle and feed per tooth on amplitude *Sz* parameters for cutting speed of 55 m/min; (**b**) influence of helix angle and cutting speed on amplitude *Sz* parameter for feed per tooth of 0.06 mm; (**c**) influence of feed per tooth and cutting speed on amplitude *Sz* parameter for helix angle of $40^{{}^{\circ}}$.

Figure 12 presents a comparable analysis for the coated tool. Figure 12a illustrates that an increase in helix angle and feed per tooth (*f_z_*) for constant cutting speed results in elevated *Sz* values, as indicated by the darker regions on the contour map. Figure 12b illustrates that an increase in cutting speed at elevated helix angles for constant feed per tooth culminates in diminished *Sz* values relative to the uncoated tool. This phenomenon can be attributed to the coating’s capacity to mitigate friction. The contour map in Figure 12b reveals a notable decline in *Sz* at elevated helix angles, a phenomenon that was not discernible in the uncoated tool. Figure 12c illustrates the impact of feed per tooth (*f_z_*) and cutting speed for a constant helix angle on *Sz* values. The contour map reveals that the coating enables the attainment of relatively low *Sz* values at elevated speeds and feed per tooth (*f_z_*), as indicated by the lighter shades in comparison to the uncoated tool.

In conclusion, in order to achieve the optimal *Sz* values (i.e., the lowest surface roughness), it is preferable to utilize the coated tool, particularly at higher cutting speeds and feed per tooth (*f_z_*) and a larger helix angle. The coating stabilizes the process and limits the increase in *Sz*, even under demanding machining conditions. Therefore, in order to minimize *Sz*, it is recommended to use a coated tool with a large helix angle, moderate cutting speed, and moderate feed per tooth (*f_z_*).

The findings of this study demonstrate significant correlations among cutting speed, feed per tooth, and helix angle and their influence on the surface roughness of NiTi alloys. These findings contribute to the advancement of current knowledge on the machining of pseudoelastic and shape memory materials, extending beyond existing studies on hard-to-machine materials such as Ti-6Al-4V and nickel-based superalloys.

The results demonstrated that higher cutting speeds led to improved surface roughness, a trend commonly observed in the machining of difficult-to-machine materials. This is typically attributed to the reduced time of interaction between the tool and the workpiece, which results in the formation of smoother chips and a reduction in the friction-induced surface irregularities. While such improvements were previously documented for materials such as Ti-6Al-4V, the current study identifies a heightened sensitivity of NiTi alloys to variations in cutting speed [14]. This is likely due to their unique pseudoelastic behavior and susceptibility to localized thermal effects during machining.

Conversely, an increase in feed per tooth was observed to result in a deterioration in surface roughness, predominantly due to an enhancement of the chip thickness and elevation of cutting forces, which in turn gave rise to a more pronounced manifestation of surface irregularities. This observation is consistent with trends observed in nickel-based superalloys, where elevated feed per tooth was shown to exacerbate mechanical and thermal stresses [19]. However, the pronounced work-hardening behavior of NiTi alloys introduces a novel challenge, whereby these effects are amplified and the necessity for precise feed rate control during the machining is underscored.

The inclination angle of the cutting edge was identified as the most influential parameter, with larger angles resulting in smoother surfaces due to improved chip evacuation and reduced friction. This behavior is consistent with prior studies on materials like Inconel 718 [31], where optimized tool geometries were shown to enhance the surface finish.

## 4. Conclusions

This study undertook a comprehensive examination of the impact of the machining parameters, specifically the feed per tooth, cutting speed and helix angle, on cutting force (*F*) and surface roughness (*Sa*, *Sz*) when milling NiTi alloys. The study focused on comparing coated and uncoated tools. The findings demonstrated that the feed per tooth (*f_z_*) exerts the most significant influence on cutting force (*F*) for both tool types. This is due to the fact that higher feed per tooth (*f_z_*) results in increased tool loading, which in turn leads to an intensification in the material volume engaged with each tooth. Nevertheless, an increase in helix angle ($\alpha_{el}$) and cutting speed (*v_c_*) was found to be effective in reducing the cutting force, due to a more distributed loading on the cutting edge and thermal softening of the workpiece material. This reduction in force was particularly evident for the coated tool, where the coating served to reduce friction, thereby enabling the tool to maintain lower cutting forces under higher machining parameters.

The analysis of the surface roughness indicated that the coated tool demonstrated superior properties, providing lower roughness values across a wider range of cutting conditions. The optimal surface quality was achieved through the combination of high cutting speed, large helix angle, and moderate feed per tooth (*f_z_*). Furthermore, the coating augmented the tool’s capacity to stabilize the process at elevated speeds and feeds, thereby reducing both the cutting force and the surface roughness, even in challenging machining environments. In contrast, the uncoated tool exhibited higher cutting forces and greater surface roughness due to increased friction and the absence of a protective layer, which resulted in greater resistance during cutting. This research highlights the superiority of coated tools for the high-efficiency milling of NiTi alloys, particularly in applications that require precise control over the cutting forces and the surface quality. The study recommends the use of coated tools with an optimized set of parameters, including high cutting speed ($v_{c}=35÷45\;m/min$), large helix angle $\left(\alpha_{el}=40{}^{\circ}\right)$, and controlled feed per tooth ($f_{z}=0.02\;mm/tooth$), to extend tool life and achieve superior surface finishes in precision machining tasks involving NiTi alloys.

This study presents a novel and original contribution to the field of precision machining, offering a comprehensive examination of the influence of coating and optimized machining parameters on cutting force and surface roughness in the milling of NiTi alloys. NiTi alloys, renowned for their shape memory properties and high biocompatibility, present a significant challenge in the machining process due to their inherent hardness, resilience, and propensity to generate high cutting forces and tool wear. This research not only demonstrates the advantages of utilizing coated tools to reduce friction and wear under challenging circumstances but also elucidates the optimal parameter ranges for achieving minimal surface roughness and cutting force. The precise interplay between feed per tooth, helix angle, and cutting speed, as explored in this study, provides a basis for further advancements in machining efficiency and surface quality. The findings have significant practical implications for industries where NiTi alloys are extensively used, such as biomedical device manufacturing, aerospace, and robotics, where precise surface quality and low cutting forces are essential. In the context of biomedical applications, the ability to achieve a surface with minimal imperfections and optimal smoothness is of paramount importance for components that interact with biological tissues. The study offers valuable insights into the optimal machining configurations for NiTi alloys, particularly the benefits of using coated tools. These insights provide useful guidance for enhancing production efficiency and component quality in precision manufacturing. This research not only advances the knowledge of NiTi alloy machining but also contributes to the broader objective of improving manufacturing practices for high-performance, biocompatible materials.

## Figures and Tables

**Table 1:** Milling parameters.

Parameters	Levels		
	1	2	3
Helix angles $\alpha_{el}$ (°)	20	30	40
Feed per tooth $f_{z}\;$(mm/tooth)	0.02	0.04	0.06
Cutting speed $v_{c}\;$(m/min)	35	45	55

**Table 2:** Values of the cutting parameters $\alpha_{el},\;f_{z},$ and $v_{c}$ for NiTi alloy, selected by means of the L9 Taguchi orthogonal array.

No.	$\alpha_{el}$ (°)	$f_{z}$ (mm/tooth)	$v_{c}$ (m/min)
1	20	0.02	35
2	20	0.04	45
3	20	0.06	55
4	30	0.02	45
5	30	0.04	55
6	30	0.06	35
7	40	0.02	55
8	40	0.04	35
9	40	0.06	45

**Table 3:** Cutting force components.

No.	$\alpha_{el}$ (°)	$f_{z}$ (mm/tooth)	$v_{c}$ (m/min)	Uncoated Carbide End Mills					Coated Carbide End Mills				
				*F_f max_* (N)	*F_r max_* (N)	*F_t max_* (N)	*F* (N)	*F_S/N* (db)	*F_f max_* (N)	*F_r max_* (N)	*F_t max_* (N)	*F* (N)	*F_S/N* (db)
1	20	0.02	35	120.9	23.4	188.3	255.4	−48.14	124.9	19.9	201.2	237.7	−49.15
2	20	0.04	45	220.0	62.9	310.8	397.4	−51.98	221.2	61.7	314.9	389.7	−52.18
3	20	0.06	55	405.3	110.5	580.9	716.8	−57.11	310.9	93.8	431.5	540.0	−55.90
4	30	0.02	45	94.9	35.6	221.4	243.5	−47.73	75.4	44.2	119.9	148.4	−46.52
5	30	0.04	55	138.2	83.3	212.1	266.5	−48.51	115.1	71.1	170.7	217.8	−49.52
6	30	0.06	35	188.6	111.0	298.8	370.4	−51.37	151.7	99.6	204.0	273.0	−51.57
7	40	0.02	55	86.9	100.0	149.6	199.8	−46.01	71.6	80.0	95.4	143.6	−46.21
8	40	0.04	35	133.6	150.0	186.2	273.9	−48.75	120.3	131.8	128.9	220.1	−47.54
9	40	0.06	45	142.2	163.7	244.6	326.9	−50.29	144.5	157.4	144.9	258.2	−51.29

**Table 4:** Surface texture parameters 3D according to ISO 25178.

No.	$\alpha_{el}$ (°)	$f_{z}$ (mm/tooth)	$v_{c}$ (m/min)	Uncoated Carbide End Mills				Coated Carbide End Mills			
				*Sz* (μm)	*Sa* (μm)	*Sa_S/N* (dB)	*Sz_S/N* (dB)	*Sz* (μm)	*Sa* (μm)	*Sa_S/N* (dB)	*Sz_S/N* (dB)
1	20	0.02	35	7.93	0.308	10.2290	−17.9855	5.12	0.374	8.5426	−14.1854
2	20	0.04	45	4.61	0.311	10.1448	−13.2740	11.50	0.983	0.1489	−21.2140
3	20	0.06	55	7.66	0.592	4.5536	−17.6846	6.17	0.523	5.6300	−15.8057
4	30	0.02	45	5.51	0.514	5.7807	−14.8230	3.41	0.301	10.4287	−10.6551
5	30	0.04	55	6.38	0.738	2.6389	−16.0964	4.17	0.393	8.1121	−12.4027
6	30	0.06	35	8.24	0.656	3.6619	−18.3185	5.09	0.415	7.6390	−14.1344
7	40	0.02	55	3.63	0.406	7.8295	−11.1981	5.40	0.505	5.9342	−14.6479
8	40	0.04	35	25.10	2.470	−7.8539	−27.9935	4.50	0.529	5.5309	−13.0643
9	40	0.06	45	12.50	1.290	−2.2118	−21.9382	6.85	0.602	4.4081	−16.7138

## Data Availability

The original contributions presented in this study are included in the article. Further inquiries can be directed to the corresponding author.

## References

[1] Dash B., Das M., Mahapatra T.R., Mishra D. (2019). A concise review on machinability of NiTi shape memory alloys. Mater. Today Proc..

[2] Kowalczyk M. (2019). Cutting force prediction in ball-end milling of Ni-Ti alloy. Proc. SPIE.

[3] Mohd J.J., Leary M., Subic A., Gibson M.A. (2014). A Review of Shape memory Alloy Research, Applications and Opportunities. Mater. Des..

[4] Fadlallah A., El-Bagoury N., Gad El-Rab S.M.F., Ahmed R.A., El-Ousamii G. (2014). An overview of NiTi shape memory alloy: Corrosion resistance and antibacterial inhibition for dental application. J. Alloys Compd..

[5] Saoud B.F., Korkmaz M.E. (2022). A Review on Machinability of Shape Memory Alloys Through Traditional and Non-Traditional Machining Processes: A Review. Manuf. Technol. Appl..

[6] Weinert K., Petzoldt V. (2004). Machining of NiTi based Shape Memory Alloys. Mater. Sci. Eng..

[7] Altas E., Gokkay H., Ozkan D. (2020). Investigation of the Effects of Machining Parameters on Tool Life and Surface Roughness During the Face Milling of the NiTi Shape Memory Alloy with Uncoated Tools. Preprints.

[8] Kaya E., Kaya I. (2020). Tool wear progression of PCD and PCBN cutting tools in high speed machining of NiTi shape memory alloy under various cutting speeds. Diam. Relat. Mater..

[9] Shizuka H., Sakai K., Yang H., Sonoda K., Nagare T., Kurebayashi Y., Hayakawa K. (2020). Difficult cutting property of NiTi alloy and its mechanism. J. Manuf. Mater. Process..

[10] Altas E., Altin Karatas M., Gokkaya H., Akinay Y. (2021). Surface Integrity of NiTi Shape Memory Alloy in Milling with Cryogenic Heat Treated Cutting Tools under Different Cutting Conditions. J. Mater. Eng. Perform..

[11] Kowalczyk M. (2024). Analysis of Cutting Forces and Geometric Surface Structures in the Milling of NiTi Alloy. Materials.

[12] Altas E., Erkan O., Ozkan D., Gokkaya H. (2022). Optimization of Cutting Conditions, Parameters, and Cryogenic Heat Treatment for Surface Roughness in Milling of NiTi Shape Memory Alloy. J. Mater. Eng. Perform..

[13] Altas E., Gokkaya H., Karatas M.A., Ozkan D. (2020). Analysis of surface roughness and flank wear using the Taguchi method in milling of NiTi shape memory alloy with uncoated tools. Coatings.

[14] Kaya E., Kaya İ. (2023). The effect of cutting tool and cutting speed on the surface integrity and functional properties in milling of NiTi shape memory alloys. J. Fac. Eng. Arch..

[15] Wang G., Liu Z., Huang W., Wang B., Niu J. (2019). Influence of cutting parameters on surface roughness and strain hardening during milling NiTi shape memory alloy. Int. J. Adv. Manuf. Technol..

[16] Gao L., Wang J., Huo H., Wang G., Huang W., Zhou X., Wang Z. (2024). Residual height of surface topography in milling nickel-titanium shape memory alloy using a small-diameter cutter. Mater. Lett..

[17] Zailani Z.A., Mativenga P.T. (2023). Machinability of nickel-titanium shape memory alloys under dry and chilled air cutting conditions. Int. J Adv. Manuf. Technol..

[18] Wang H., Wang B., Liu Z., Li Z., Liu H., Song Q. (2023). A novel dealloying assisted machining method to improve the machinability of NiTi alloy as a typical high toughness difficult-to-machine material. Mater. Des..

[19] Wang H., Wang B., Liu Z., Li Y., Zhang R., Zhao J. (2024). Effects of dealloying surface modification on machinability improvement of NiTi alloy during micro-machining process. Tribol. Int..

[20] Kaynak Y., Manchiraju S., Jawahir I.S. (2015). Modeling and simulation of machining-induced surface integrity characteristics of NiTi alloy. Proc. CIRP.

[21] Kaya E., Kaya I. (2019). A review on machining of NiTi shape memory alloys: The process and post process perspective. Int. J. Adv. Manuf. Technol..

[22] Wan M., Feng J., Zhang W.H., Yang Y., Ma Y.C. (2017). Working mechanism of helix angle on peak cutting forces together with its design theory for peripheral milling tools. J. Mater. Process. Technol..

[23] Sur G., Motorcu A.R., Nohutçu S. (2022). Single and multi-objective optimization for cutting force and surface roughness in peripheral milling of Ti6Al4V using fixed and variable helix angle tools. J. Manuf. Process..

[24] Tien D.H., Nguyen N.T., Duc T.D. (2019). Influence of Different Cutter Helix Angle and Cutting Condition on Surface Roughness During End-Milling of C45 Steel. Int. J. Mech. Eng. Technol..

[25] Plodzien M., Burek J., Zylka L., Sulkowicz P. (2020). The influence of end mill helix angle on high performance milling process. J. Mech. Sci. Technol..

[26] Jiang R., Wang J., Gao Y., Zhu Z., Cao P. (2021). The researches concern the influence of the helix angle on the composite machining process. Comp. Adv. Mater..

[27] Zagórski I., Szczepaniak A., Kulisz M., Korpysa J. (2022). Influence of the Tool Cutting Edge Helix Angle on the Surface Roughness after Finish Milling of Magnesium Alloys. Materials.

[28] Joshi S.N., Bolar G. (2021). Influence of End Mill Geometry on Milling Force and Surface Integrity While Machining Low Rigidity Parts. J. Inst. Eng..

[29] Knápek T., Dvořáčková Š., Váňa M. (2023). The Effect of Clearance Angle on Tool Life, Cutting Forces, Surface Roughness, and Delamination during Carbon-Fiber-Reinforced Plastic Milling. Materials.

[30] Yin Q., Liu Z., Wang B., Song Q., Cai Y. (2020). Recent progress of machinability and surface integrity for mechanical machining Inconel 718: A review. Int. J. Adv. Manuf. Technol..

[31] Yin Q., Li C., Don L., Bai X., Zhang Y., Yang M., Jia D., Li R., Liu Z. (2021). Effects of Physicochemical Properties of Different Base Oils on Friction Coefficient and Surface Roughness in MQL Milling AISI 1045. Int. J. Precis. Eng. Manuf.-Green Technol..

[32] Zhao Y., Guo K., Li J., Sun J. (2021). Investigation on machinability of NiTi shape memory alloys under different cooling conditions. Int. J. Adv. Manuf. Technol..

[33] Stojanovic B., Blagojevic J., Babic M., Velickovic S., Miladinovic S. (2017). Optimization of hybrid aluminum composites wear using Taguchi method and artificial neural network. Ind. Lubr. Tribol..

[34] Kiran K., Kayacan M.C. (2019). Cutting force modeling and accurate measurement in milling of flexible workpieces. Mech. Syst. Signal Process..

[35] Ducroux E., Fromentin G., Viprey F., Prat D., D’Acunto A. (2021). New mechanistic cutting force model for milling additive manufactured Inconel 718 considering effects of tool wear evolution and actual tool geometry. J. Manuf. Process..

[36] Kowalczyk M. (2017). Application of Taguchi method to optimization of surface roughness during precise turning of NiTi shape memory alloy. Proc. SPIE.

[37] Morelli L., Grossi N., Scippa A., Campatelli G. (2021). Surface error shape identification for 3-axis milling operations. Procedia CIRP.

[38] Kaynak Y., Huang B., Karaca E.H., Jawahir S.I. (2017). Surface Characteristics of Machined NiTi Shape Memory Alloy, The Effects of Cryogenic Cooling and Preheating Conditions. J. Mater. Eng. Perform..

